# The role of artificial intelligence in diagnostic neurosurgery: a systematic review

**DOI:** 10.1007/s10143-025-03512-2

**Published:** 2025-04-28

**Authors:** William Li, Armand Gumera, Shrushti Surya, Alex Edwards, Farynaz Basiri, Caleb Eves

**Affiliations:** 1 Department of Ophthalmology, University of Sydney, Sydney, Australia; 2 Department of Surgery, University of Melbourne, Melbourne, Australia; 3 University of Sydney School of Medicine, Sydney, Australia; 4 Macquirie University, Sydney, Australia; 5 School of Medicine, University of Wollongong, Wollongong, Australia

**Keywords:** Neurosurgery, AI, Diagnosis, Machine learning

## Abstract

**Background:**

Artificial intelligence (AI) is increasingly applied in diagnostic neurosurgery, enhancing precision and decision-making in neuro-oncology, vascular, functional, and spinal subspecialties. Despite its potential, variability in outcomes necessitates a systematic review of its performance and applicability.

**Methods:**

A comprehensive search of PubMed, Cochrane Library, Embase, CNKI, and ClinicalTrials.gov was conducted from January 2020 to January 2025. Inclusion criteria comprised studies utilizing AI for diagnostic neurosurgery, reporting quantitative performance metrics. Studies were excluded if they focused on non-human subjects, lacked clear performance metrics, or if they did not directly relate to AI applications in diagnostic neurosurgery. Risk of bias was assessed using the PROBAST tool. This study is registered on PROSPERO, number CRD42025631040 on January 26th, 2025.

**Results:**

Within the 193 studies, neural networks (30%) and hybrid models (48.2%) dominated. Studies were categorised into neuro-oncology (52.69%), vascular neurosurgery (19.89%), functional neurosurgery (16.67%), and spinal neurosurgery (11.83%). Median accuracies exceeded 85% in most categories, with neuro-oncology achieving high diagnostic accuracy for tumour detection, grading, and segmentation. Vascular neurosurgery models excelled in stroke and intracranial haemorrhage detection, with median AUC values of 88% and 97%, respectively. Functional and spinal applications showed promising results, though variability in sensitivity and specificity underscores the need for standardised datasets and validation.

**Discussion:**

The review’s limitations include the lack of data weighting, absence of meta-analysis, limited data collection timeframe, variability in study quality, and risk of bias in some studies.

**Conclusion:**

AI in neurosurgery shows potential for improving diagnostic accuracy across neurosurgical domains. Models used for stroke, ICH, aneurysm detection, and functional conditions such as Parkinson’s disease and epilepsy demonstrate promising results. However, variability in sensitivity, specificity, and AUC values across studies underscores the need for further research and model refinement to ensure clinical viability and effectiveness.

**Supplementary Information:**

The online version contains supplementary material available at 10.1007/s10143-025-03512-2.

## Introduction

Artificial intelligence (AI) enables computers to perform tasks that typically require human cognition. In neuro-oncology, vascular, functional and spinal-neurosurgery, AI is increasingly applied to enhance diagnostic precision and assist in complex procedures where variability and risk are high. These processes, which traditionally rely on the surgeon’s intuition, experience, and learned knowledge, pose the question of how AI can replicate such intricate aspects of human intelligence without directly mirroring cognitive processes step by step.

Machine Learning (ML), a subset of AI, offers a different approach. Rather than explicitly programming a system to replicate human reasoning, ML involves training algorithms on the patient histories, scans, examination findings, and other findings. Through processes not completely transparent the AI then recognises patterns that they generalise to previously unseen patient findings.

A variety of AI approaches, each tailored to distinct neurosurgical applications, have emerged to enhance patient evaluations before, during, and after surgery.

1. Supervised Learning – This approach trains AI models on datasets such as MRI images, where it is labelled whether the scan is abnormal or normal [1, 2]. In neurosurgery, supervised learning is frequently applied in diagnostic imaging—where models are trained on thousands of labelled MRI scans to detect brain tumours. As the model undergoes iterative training, its accuracy in classifying new, unseen scans improves. Beyond classification, supervised learning is valuable for tasks that predict continuous variables, including tumour growth trajectories, patient survival rates, and the optimisation of drug dosages to enhance therapeutic outcomes. There are different types of supervised learning such as logistic regression (LR), support vector machines (SVM), neural networks (NN), deep neural networks (DNN), decision trees (DT), random forests (RF), and Naïve Bayes (NB) [3].
  LR is widely employed to predict binary outcomes [4, 5]. While the final output of LR is typically binary, the model assigns probabilities ranging between 0 and 1, indicating the likelihood of a specific event [5]. LR’s simplicity and interpretability make it an attractive option for neurosurgical applications, such as predicting the risk of postoperative complications or classifying patients based on tumour presence. However, the accuracy of LR models require large datasets to ensure robust predictions. Support Vector Machines (SVMs) are highly flexible in modelling complex relationships but can be prone to overfitting [6] where the model learns the training data too thoroughly, capturing patterns that do not generalize to unseen scans or data [7]. Despite this they are valuable in neurosurgery, particularly for rare conditions where data availability is often sparse. NNs often predict things better than linear models [3, 8, 9] such as DTs, which are more so favoured for their ease of training, interpretability. DNNs are an expanded version of NNs which incorporates many more layers [3]. DNNs are amongst the most complex models in current use involving multiple layers [3, 10]. Even though they require significant amounts of data, they deliver significantly more predicative performance than conventional models [3, 11]. DTs work by dividing data through a series of yes/no questions or threshold-based splits[2]. Each split continues until the data is fully classified or certain stopping conditions are met [12]. Random Forest (RF) builds many DTs and combines their results to synthesize better predictions [13, 14]. Additionally, when each tree splits, it only considers a random selection of features [14, 15]. This randomness makes RF particularly useful in diagnostic neurosurgery for predicting tumour types based on imaging data and patient history. Naive Bayes (NB) operates by assuming that all data features are independent [16]. However, in real-world situations, this assumption often fails because many features are interconnected [16]. Despite this, NB is commonly used in situations where its speed, simplicity, and ability to manage smaller datasets are advantageous [17].

2) Unsupervised learning—In contrast, unsupervised learning involves training AI models on datasets that lack labelled outputs[2]. For example, an AI system may analyse large collections of brain scans without any indication of whether each scan is normal or indicative of a tumour. The model autonomously identifies patterns and clusters similar data points. This technique can reveal novel glioma subtypes or detect subtle anomalies that may not be immediately apparent to radiologists or neurosurgeons.
3) Reinforcement learning—This is a machine learning approach in which an AI receives rewards for correct outcomes and penalties for undesirable ones. This iterative process allows the AI to refine its performance over time. For instance, in a medical imaging context the AI may receive
  + 10 points for accurately diagnosing a brain tumour
  − 5 points for missing a tumour (false negative) and
  − 5 points for incorrectly identifying a tumour where none exists (false positive)
  By reinforcing correct decisions and penalizing mistakes, the AI progressively improves its diagnostic accuracy.
4) Semi-supervised learning—This method bridges the gap between supervised and unsupervised learning. In the context of brain tumour detection, obtaining large volumes of labelled MRI scans can be time-consuming and expensive. Semi-supervised learning allows the AI to leverage a limited set of labelled scans, using them to infer patterns in the broader, unlabelled dataset. This approach can enhance detection accuracy by enabling the AI to generalise better, identifying nuanced tumour characteristics even with minimal labelled input. It is particularly useful for rare tumour types or subtle pathological variations that may be underrepresented in labelled datasets.

While the input (e.g., MRI images) and output (e.g., diagnosis) are observable, the specific pathways and computations that lead to these outputs can be complex and not completely transparent.

## Research question

*"In patients undergoing diagnostic evaluation for neurosurgical conditions, how does the use of AI-assisted diagnostic tools perform in metrics of accuracy, sensitivity, and specificity?"*

## Materials and methods

### Search strategy

The search was conducted in September and October of 2024.

A comprehensive search strategy was employed using a combination of domain-specific terms (e.g., neurosurgery, spine surgery) and methodological terms (e.g., machine learning, neural networks). MeSH terms were utilized to ensure complete coverage of indexed articles. This dual-category approach was designed to capture studies at the intersection of neurosurgical applications and AI methodologies, minimizing the risk of omitting relevant literature.

We systematically searched PubMed, Cochrane Library, China National Knowledge Infrastructure (CNKI), and Embase for studies published between January 2020 and January 2025. ClinicalTrials.gov was also queried for ongoing or completed trials. Search terms included combinations of “Artificial Intelligence,” “Machine Learning,” “Deep Learning,” and “Diagnostic Neurosurgery,” alongside condition-specific terms such as “Spine Pathology,” “Brain Tumours,” “Epilepsy,” and “Cerebrovascular.” Boolean operators (AND/OR) were used to refine the search. Example queries included: “Artificial Intelligence AND Diagnostic Neurosurgery” and “Deep Learning AND Brain Tumours.” Table 1 shows the keywords and MeSH terms.

**Table 1:** Keywords and MeSH terms used in search strategy

	Domain- specific terms	Methodological terms
Keywords	- Spine surgery - Neurosurgery - Intraoperative/perioperative procedure - Preoperative	- Machine learning - Neural networks - Deep neural networks - Convoluted neural networks - Random forest - Predictive model - Logistic regression - Supervised learning - Unsupervised learning - Support Vector Machine
MeSH terms	- Neurosurgery - Clinical decision supports - Neurosurgical procedures	- Machine learning - Unsupervised machine learning

### Inclusion criteria

1. Studies involving AI in diagnostic neurosurgery.
2. Studies reporting quantitative performance metrics (e.g., accuracy, sensitivity, specificity).
3. English and Chinese language publications.
4. Original research relevant to the research question.
5. Studies focused on diagnosing spinal pathologies, epilepsy, neuro-oncology, or cerebrovascular conditions.

### Exclusion criteria

1. Studies unrelated to diagnostic neurosurgery
2. Studies lacking measurable outcomes or performance metrics.
3. Studies published prior to 2020
4. Studies using unvalidated or theoretical AI models without clinical application.
5. Retracted publications
6. Conference papers, abstracts, commentaries, and letters
7. Studies without original data
8. Studies involving non-human subjects

### Screening

**Fig. 1:**
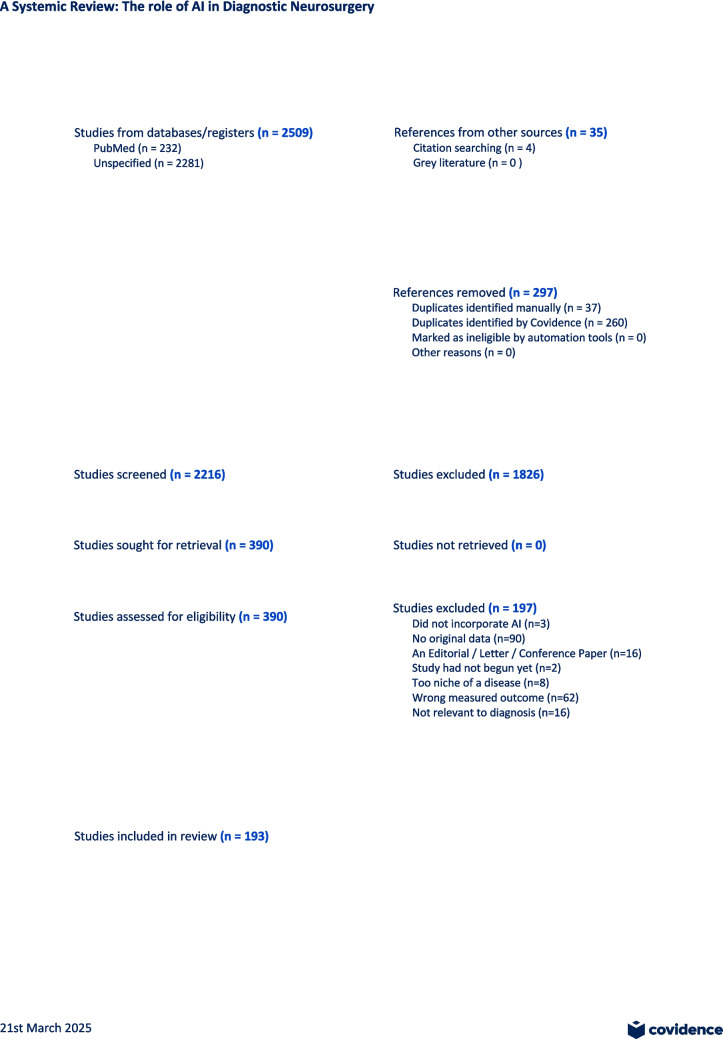
PRISMA Diagram

Titles and abstracts of all retrieved studies (n = 2513) were screened for relevance against the inclusion and exclusion criteria using Covidence software. There were 156 duplicates removed in total, 129 of those were automatically removed through the Covidence software and 29 were manually removed. 1752 studies were screened out during the title and abstract screening, where 371 studies underwent full-text review. Exclusion reasons for full-text articles were systematically documented using a PRISMA flow diagram. 371 studies underwent full text screening of which 185 studies were screened out, and 193 eligible studies underwent data extraction. Screening was conducted independently by two reviewers at both the title/abstract and full-text stages. Discrepancies were resolved through discussion with input from a third reviewer when necessary to reach consensus. Risk of bias was also assessed for these remaining studies using the Prediction model Risk of Bias Assessment Tool (PROBAST). This is highlighted in Fig. 1

## Results

### Data extraction

193 articles were extracted for their data through 2 independent reviewers in Covidence with discrepancies resolved with a third reviewer.

The following variables were collected from each study:

1. **Study Details**: Title of paper, name of first author, country the study was conducted in, aim of the study, and study design.
2. **AI Model Information**: Name and models used or developed, architecture, and the training technique used eg. Supervised, unsupervised, transfer learning.
3. **Dataset Information:** The source of the data, the timeframe in which the data was collected, the n of training, testing and validation set when applicable.
4. **Target Disease:** The disease that the AI detected, the subtype of disease, and the type of dataset eg. Scans, blood results, analysis of CSF
5. **Performance Metrics**: Accuracy, sensitivity, specificity, AUC, Intraclass Correlation Coefficient (ICC), false positives and negatives, F1 score where available.

The data analysis was conducted by 2 independent analysts where discrepancies were reviewed by the 1 st Author. The quantitative analysis software or method was at the discretion of each analyst. Most studies reported sensitivity, specificity, accuracy, and AUC. Studies that provided only some of these metrics were still included. Unreported values were labelled as “NR” and accounted for in the final calculations.

### Publication of AI in diagnostic neurosurgery over time

**Fig. 2:**
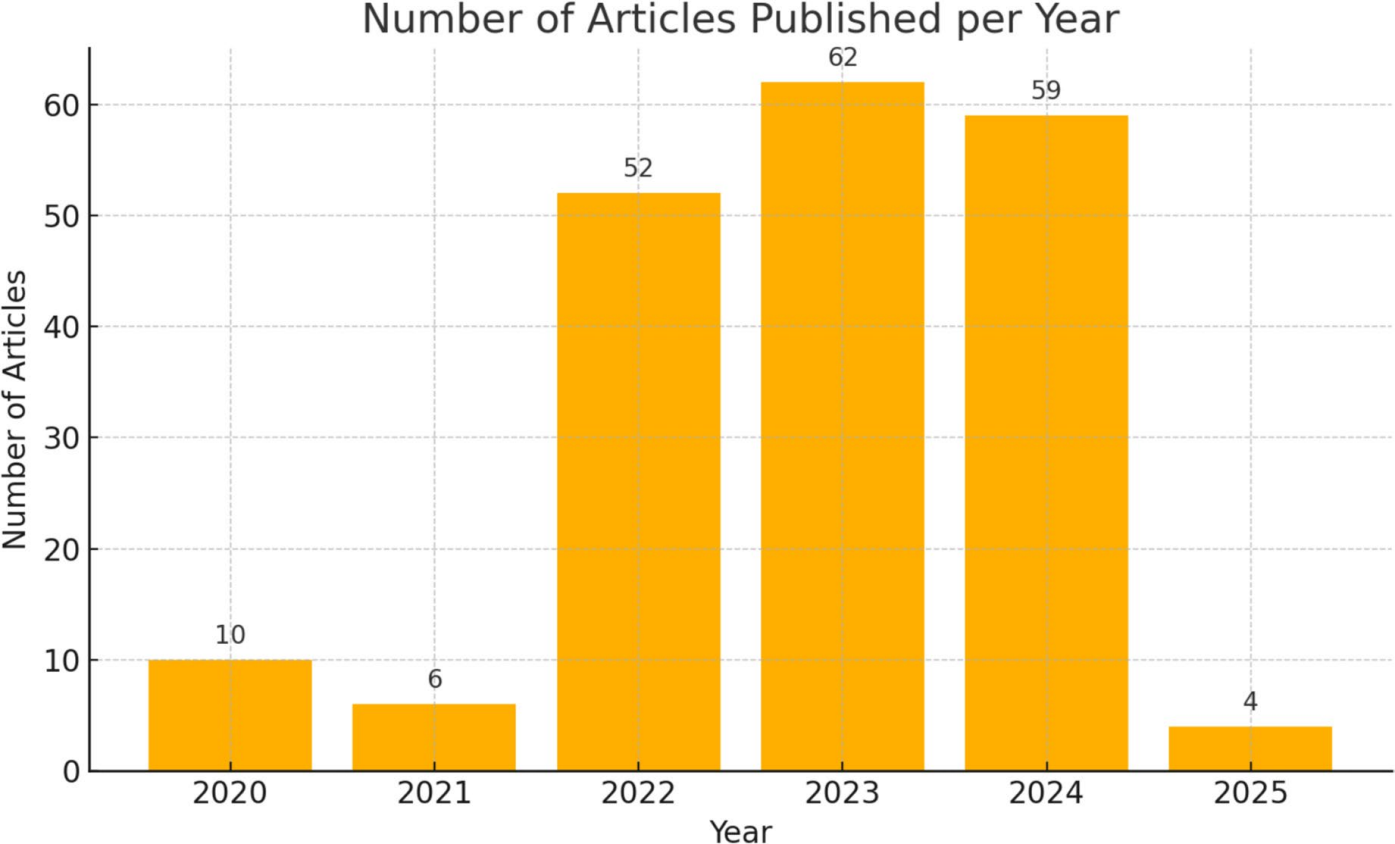
Number of AI Publications over time

The number of publications applying AI to diagnostic neurosurgery has drastically increased over the years. Of our final group of 193 articles, 10 of them were published in 2020, 6 in 2021, 52 in 2022, 62 in 2023, 59 in 2024, and 4 in 2025. Please see Fig. 1 for more information (Fig. 2).

### Countries of published studies of AI’s role in diagnostic neurosurgery

**Fig. 3:**
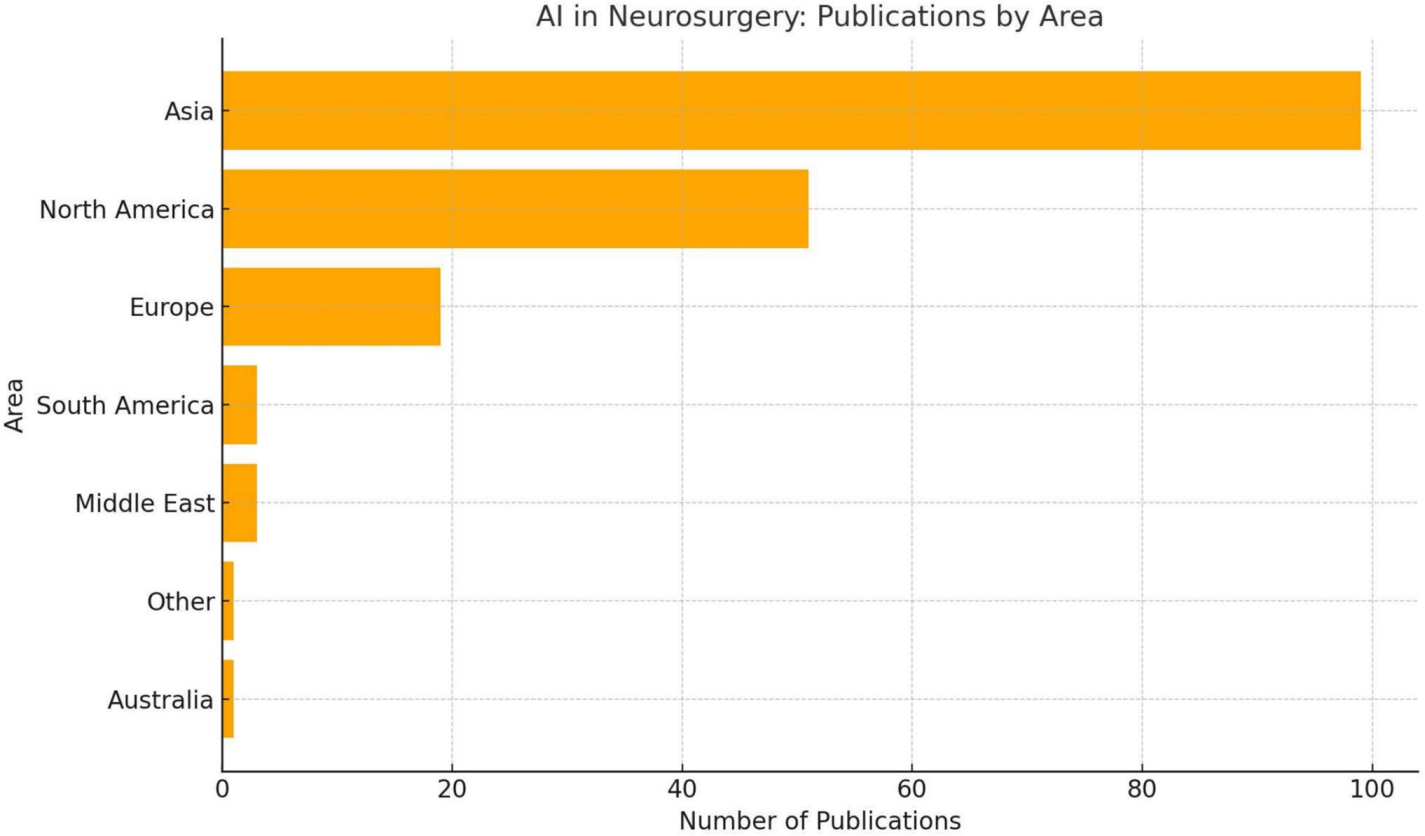
AI Publications by Region

**Fig. 4:**
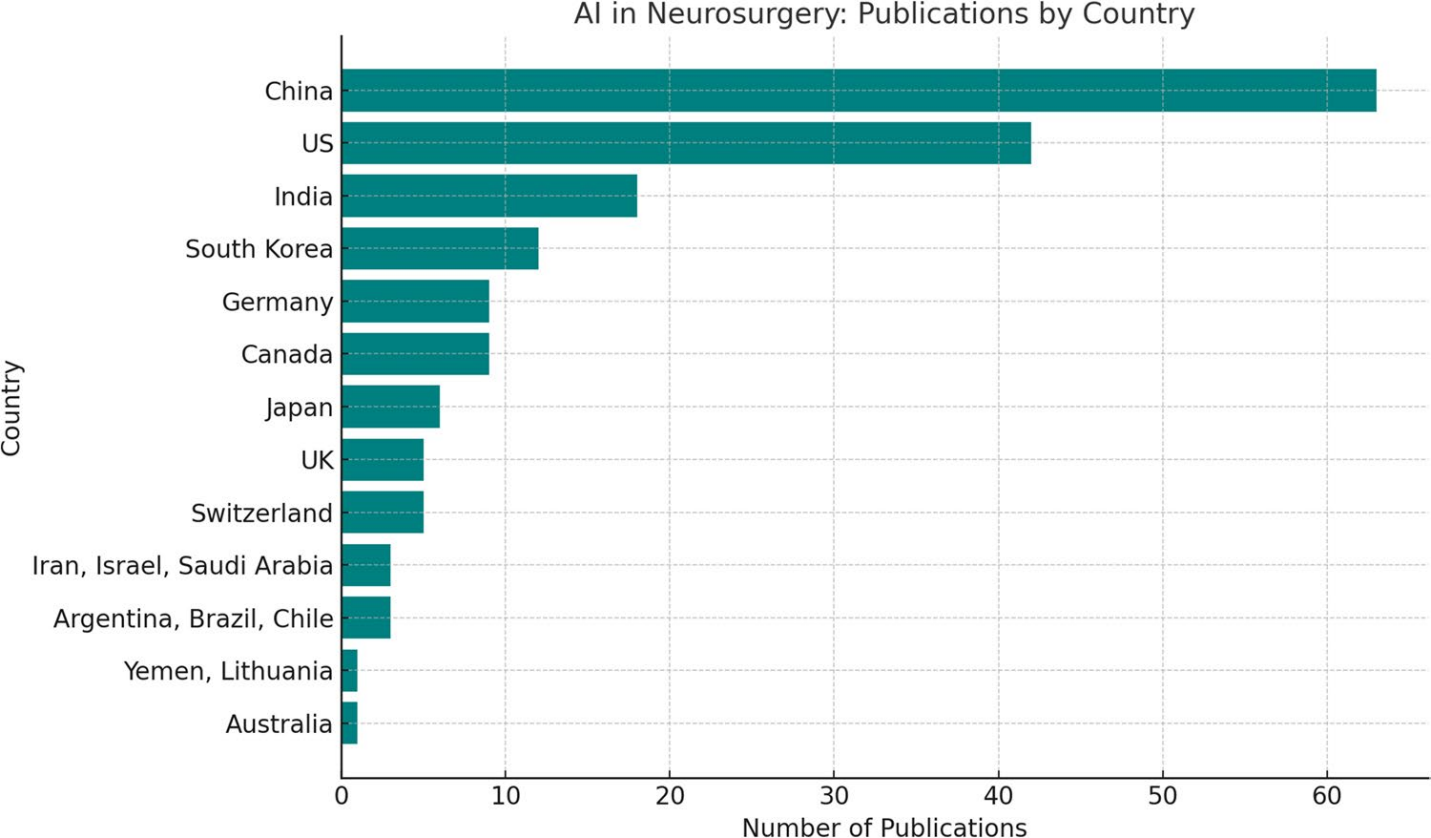
AI Publications by Country

The data on publication counts across countries reveals significant regional disparities in research output. Asia emerges as the leading contributor, with China dominating at 64 publications, followed by India [18], South Korea [12], and Japan [6]. North America also showcases robust output, driven primarily by the US [19] and Canada [9]. Europe contributes a notable share, with Germany [10], Switzerland [5], and the UK [6] among the key players. Other regions like South America and the Middle East have modest representation, with Argentina, Brazil, and Chile collectively contributing three publications, while countries like Iran, Israel, and Saudi Arabia add to the Middle East's total. Australia and other smaller contributors such as but not limited to Yemen and Lithuania represent the minimal-output category. Highlights AI development by country, whereas highlights AI development by region. These are shown in both Figs. 3 and 4.

### The type of AI models used

**Fig. 5:**
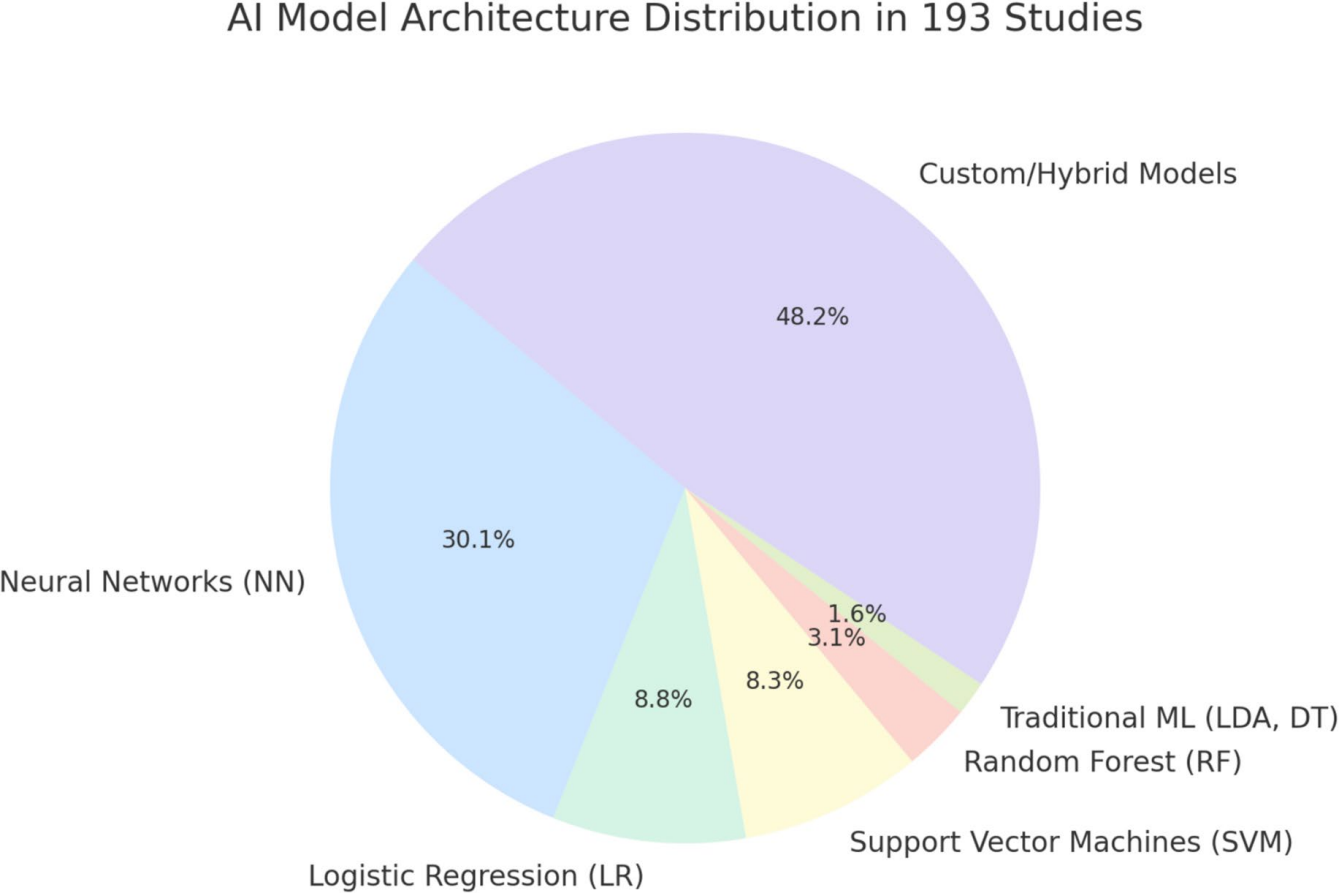
AI Model Type Distribution

Our analysis of the 193 studies revealed a diverse range of AI models applied in the research categorised into 6 underlying architectures (shown in Fig. 5):

1. Neural Networks (NN): This category, comprising 58 studies (30%), includes models such as Convolutional Neural Networks (CNNs), U-Nets, and other NN-based architectures.
2. Logistic Regression (LR): Representing 17 studies (9%), logistic regression models were primarily used for disease classification such as differentiation between Primary CNS lymphoma from GBMs.
3. Support Vector Machines (SVM): SVMs appeared in 16 studies (8%) and were applied to distinguish similar diseases such as distinguishing between brain tumours subtypes and epilepsy phenotypes.
4. Random Forest (RF): This category, accounting for 6 studies (3%), involved ensemble tree-based models.
5. Traditional Machine Learning (e.g., LDA, DT): 3 studies (2%) utilized traditional algorithms, including Linear Discriminant Analysis (LDA) and Decision Trees (DT).
6. Custom/Hybrid Models: 93 studies (48%) employed hybrid or custom architectures. These models often integrated neural networks with traditional methods or were uniquely designed for specific tasks. Examples included cascade frameworks combining segmentation and classification and hybrid pipelines leveraging CNNs and Random Forests.

Custom and hybrid models, comprising nearly half (48.2%) of the studies, highlights the surge of innovation during AI’s formative years. These models blend established techniques, such as neural networks (NNs), Random Forests (RF), or Support Vector Machines (SVMs). This diversity reflects a period of experimentation where numerous designs compete before a few iconic models eventually emerge.

### Distribution of training techniques for AI models

**Fig. 6:**
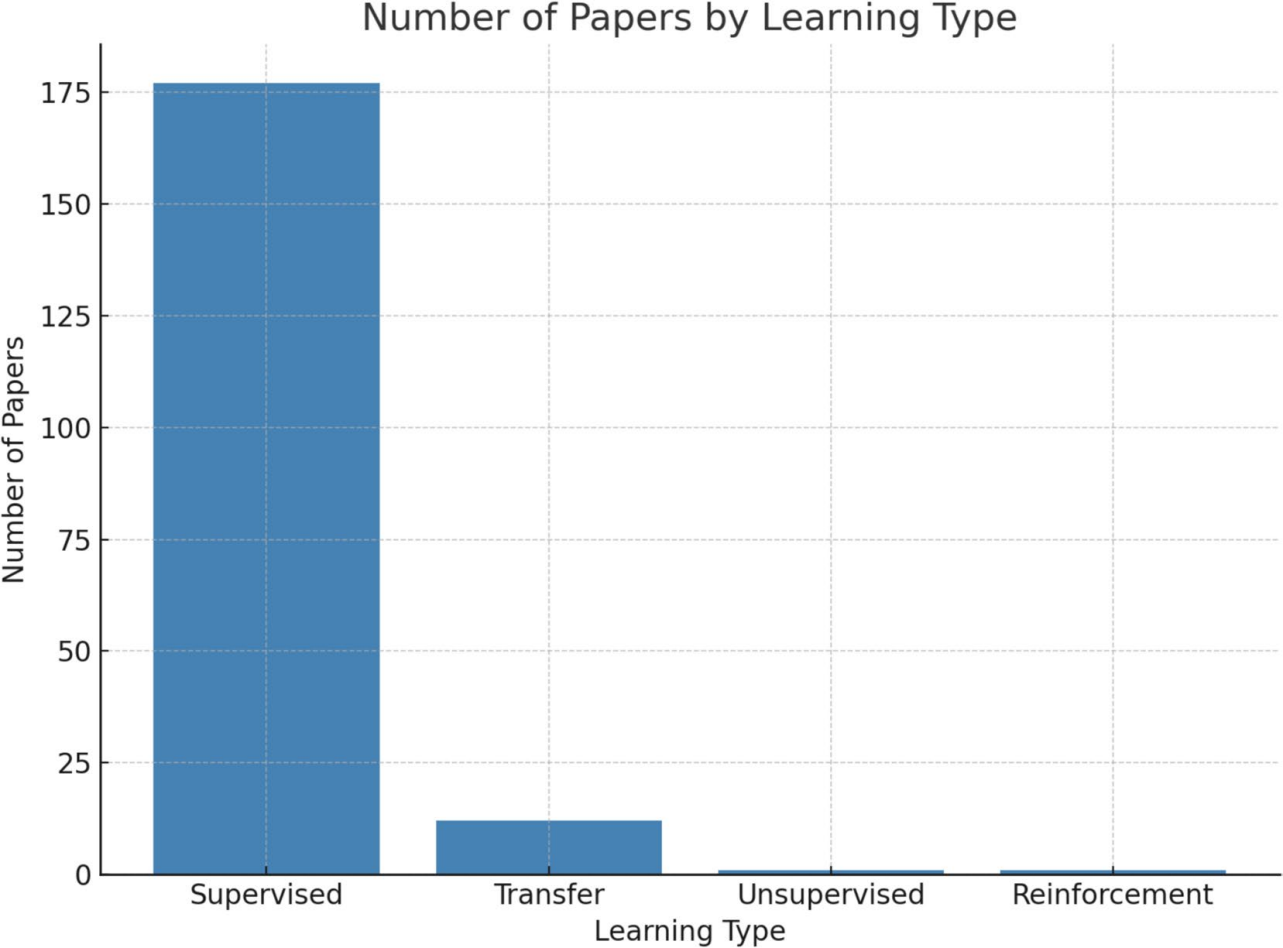
AI Distribution of Learning Types

A total of 177 papers utilized supervised learning-based approaches. Additionally, there was 1 paper employing unsupervised learning, 12 papers leveraging transfer learning, and 1 paper utilizing reinforcement learning. (Shown in Fig. 6).

### Types of input data used by AI models

**Fig. 7:**
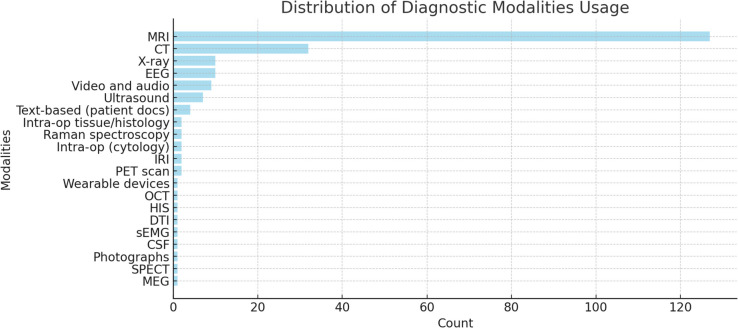
Distribution of Diagnostic Modalities

Within these studies, MRI was the most used modality [20], followed by CT [21], Ultrasound [7], and X-ray/EEG [10]. Video/audio data [9] and text-based documents [4] are moderately used, while others like Raman spectroscopy, PET, SPECT, and MEG range between 1–4. Figure 7 highlights this.

### Distribution of study focus across neurosurgical categories

**Fig. 8:**
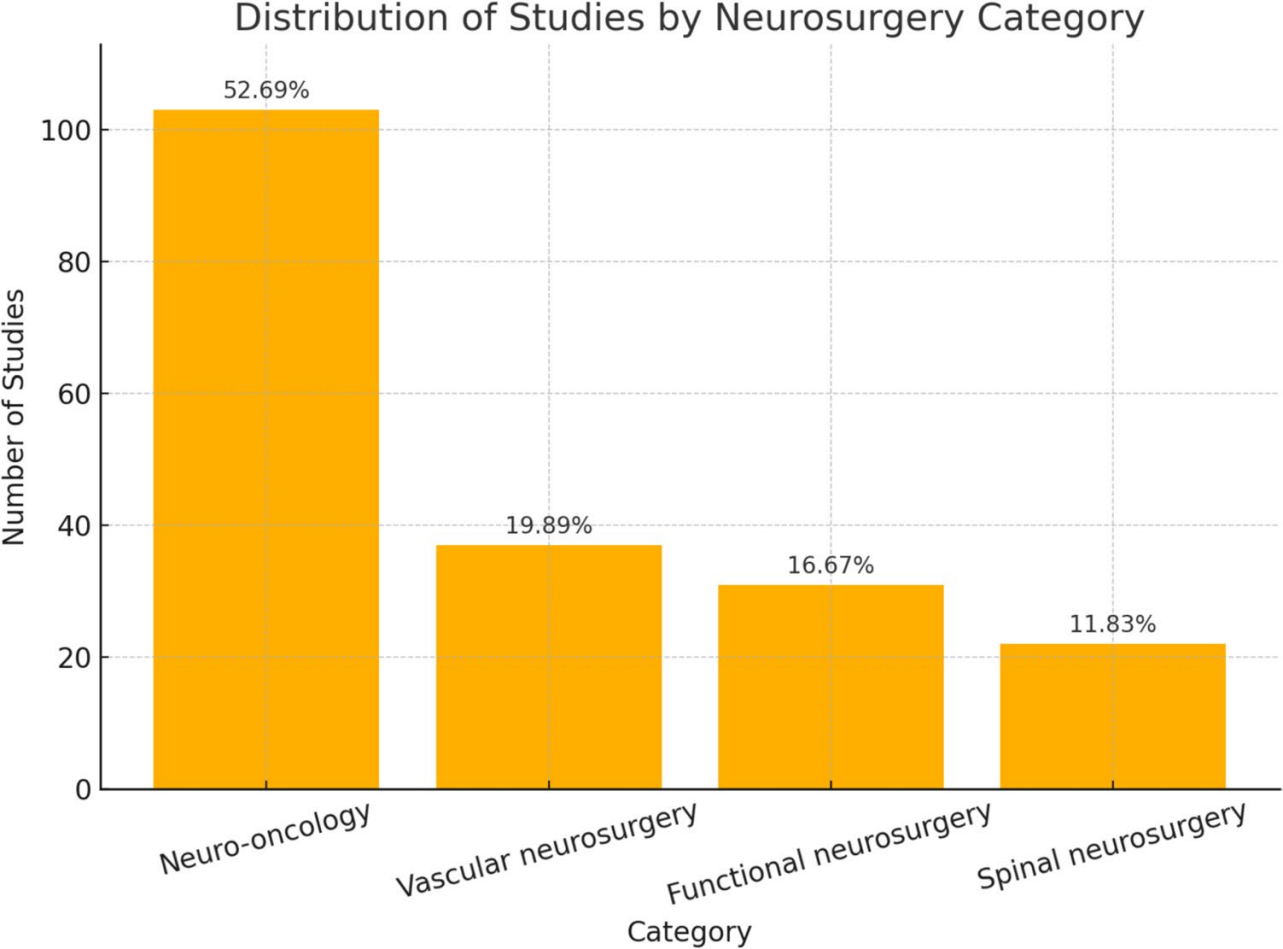
Distribution of AI in Sub-Specialties of Neurosurgery

Most of the 193 studies commonly fell into 4 principal categories (as shown in Fig. 8).

1. Neuro-oncology (n = 103, 52.69%)
2. Vascular neurosurgery (n = 37, 19.89%)
3. Functional neurosurgery (n = 31, 16.67%)
4. Spinal neurosurgery (n = 22, 11.83%)

#### Performance metrics used to assess AI models

**Fig. 9:**
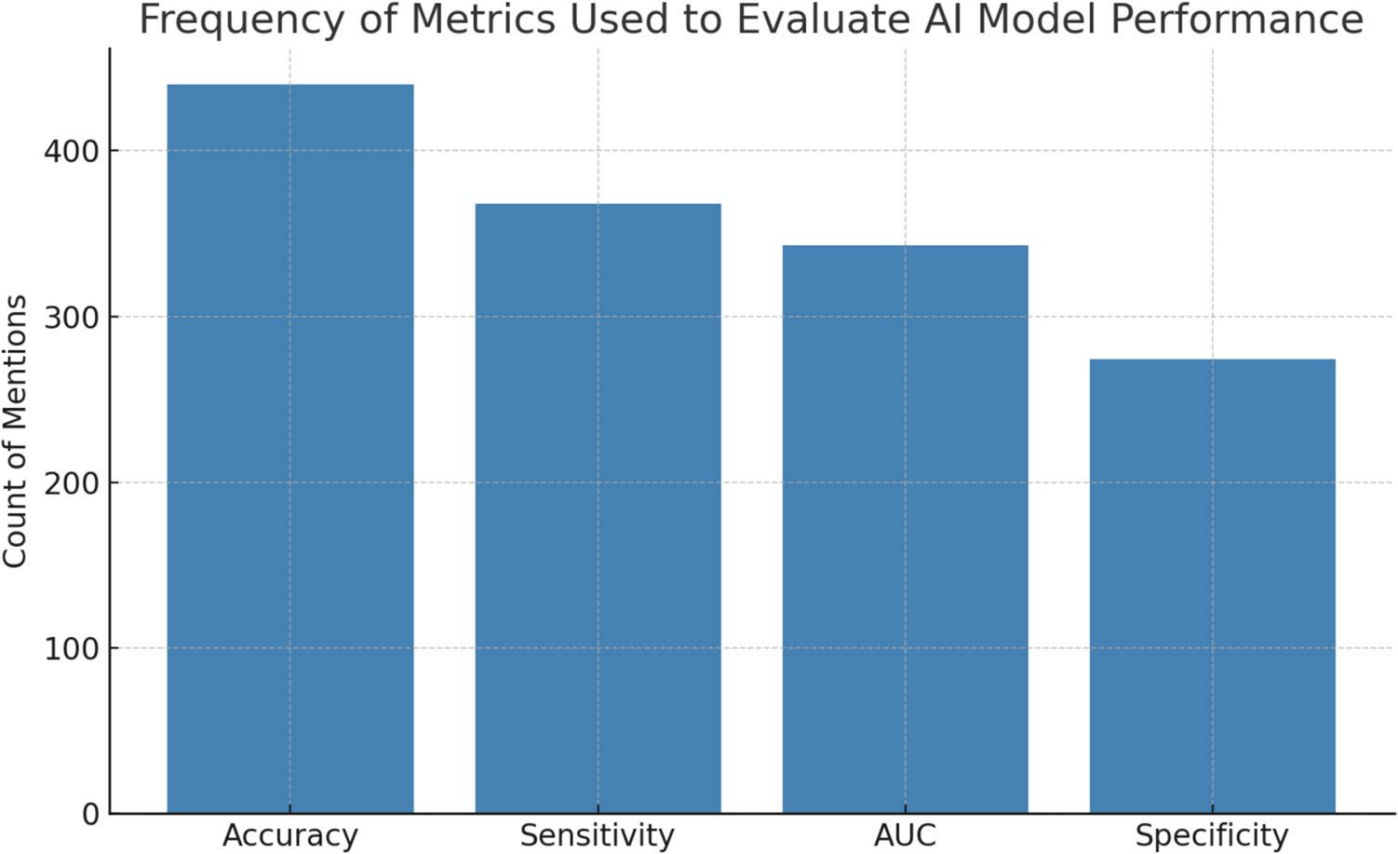
Frequency of Metrics used to Evaluate AI Performance

The most used metrics to evaluate AI model performance were accuracy, sensitivity, specificity, and AUC. Accuracy was most frequently documented (442 mentions), followed by sensitivity (370 mentions), and AUC (345 mentions). Specificity was reported in 276 instances, emphasising its role in evaluating false positives. Metrics such as precision, FP/pt, recall and F1 score were less commonly reported. Such data is represented by Fig. 9.

### Accuracy of the AI models

#### Neuro-oncology

105 of the neuro-oncology studies encompassed a diverse range of neuro-oncological conditions and diagnostic objectives, which we categorised into distinct groups for clarity and relevance. These include:

1. Classification between normal and abnormal tissue

These studies involved differentiating normal tissue from low-grade gliomas in paediatric patients and differentiating normal brain tissue from low-grade gliomas intra-operatively. Other applications include differentiating true progression from radio necrosis in brain metastases and distinguishing true progression from pseudo progression in GBMs. A total of 8 papers were reviewed in this area with 16 AI models tested.

The accuracy, specificity, sensitivity, and area under the curve (AUC) varied across studies. Table 2 shows the calculated statistical summaries:

- Accuracy: The median accuracy was 85%, with an average of 81%, and a range of 37%
- Specificity: The median specificity was 83%, with an average of 79%, and a range of 50%
- Sensitivity: The median sensitivity was 78.3%, with an average of 76.47%, and a range of 68.8%
- AUC: The median AUC was 77%, with an average of 77%, and a range of 34%

**Table 2:** AI Performance in Classification between Normal and Abnormal Tissue

Model number in spreadsheet	Study Purpose	First Author	Accuracy	Specificity	Sensitivity	AUC
3	Use of RS and ML to differentiate normal brain tissue from tumour in pediatric pts	Jabarkheel	89.80%	92.30%	84.90%	94%
27	To evaluate the effectiveness of VRR analyser in intraoperative detection of tumour boundary identification	Zhang	95.50%	50%	99.10%	75.50%
40	To apply a radiomics based AI to differentiate between true progression and radionecrosis	Peng	NR NR	86.67% 86.97%	65.38% 97%	81% 79%
47	To develop an AI to distinguish between true progression from pseudo progression in GBM	Yadav	85%	100%	70%	84%
66	Using an AI model to distinguish between glioma recurrence from pseudo progression	Fang-Xiong Fu	88%	50%	100%	90%
81	To investigate the use of ML in distinguishing radiation necrosis from tumours in pts undergone stereotactic radiosurgery	Basree	NR NR NR NR NR	NR NR NR NR NR	NR NR NR NR NR	73% 69% 64% 64% 68%
88	To develop an AI to differentiate between recurrent GBM and radiation necrosis from chemotherapy	Park	80.50% 78%	82.90% 87%	78.30% 66.7%	90% 80%
104	to develop and validate a rapid intraoperative molecular diagnostic method for glioma using ultrasound radio-frequency	Xie	85% 65.6% 58.8%	81.6% 72.6% 78.8%	89% 59.6% 31.2%	85% 72% 60%

2) Tumour Subtyping and Grading

These studies classify of tumours by grade, such as gliomas and meningiomas, and differentiating between high-grade and low-grade classifications. It also includes studies identifying molecular subtypes of diffuse gliomas using MRIs and employing diffusion kurtosis imaging (DKI) histograms to classify glioma grades. Specific examples include classifying paediatric medulloblastomas into four molecular subgroups, distinguishing between benign and malignant vertebral compression fractures, and using AI to differentiate primary, secondary, and lymphoma lesions of the temporal bone skull base. This topic was covered in 19 papers and 65 AI models or original datasets were assessed. Details are shown in table Table 3.

- Accuracy: The median accuracy was 88%, with an average of 86%, and a range of 30%.
- Specificity: The median specificity was 88%, with an average of 86%, and a range of 32.4%.
- Sensitivity: The median sensitivity was 90%, with an average of 89%, and a range of 26%.
- AUC: The median AUC was 90%, with an average of 89%, and a range of 34%.

**Table 3:** AI Performance in Tumour Subtyping and Grading

Model number in spreadsheet	First Author	Accuracy	Specificity	Sensitivity	AUC
4	Li	83% 76% 80% 74% 83% 79% 74% 69%	NR NR NR NR NR NR NR NR	NR NR NR NR NR NR NR NR	89% 84% 89% 82% 85% 77% 66% 68%
7	Chandra	95.87% 83.53% 84.24% 92.77% 93.57% 91.78%	NR NR NR NR NR NR	NR NR NR NR NR NR	NR NR NR NR NR NR
9	Yang	92.60% 88.9% 70.4%	NR 92.4% NR	NR 90% NR	93.50% 91.8% 71.8%
14	Jiang	85.70% 81.4% 82%	92% 80% 89%	77.10% 86.9% 75.4%	90.40% 89.90% 86.6%
15	Lam	88% 85% 92%	85% 83% 89%	91% 88% 94%	90% 87% 95%
16	Zhang	92.90% 92.9% 83.3% 90.5% 78.6% 81%	77.80% 77.8% 77.8% 66.7% 66.7% 88.9%	97% 97% 84.9% 97% 81.8% 78.8%	82.50% 92.3% 92.3% 87.2% 78.1% 91.4%
20	Tandel	99.06% 98.63% 98.43%	99.10% 98.57% 98.57%	99.04% 98.7% 98.33%	99% NR NR
23	You	93.10% 91.2% 76.1%	89.20% 91.9% 72.3%	95.40% 90.8% 77.6%	96.60% 93.7% 83.1%
33	Kumar	83% 82%	NR NR	NR NR	81% 82%
38	Jun	72.10%	71.70%	73.30%	77%
43	Taslicay	93.80% 92.30% 90.80%	NR NR NR	NR NR NR	96% 98% 94.5%
50	Cheng	92.50% 89.30% 89.30%	91.30% 95% 87.5%	87.10% 90% 91.7%	93% 99% 93%
54	Maskani	93.50% 90.3% 87.1%	90.30% 90.3% 87%	96.77% 90.3% 87%	98% 94% 87%
58	Usuzaki	84%	NR	NR	93%
59	Kozel	85%	NR	NR	NR
63	Schumann	98% 98% 75% 79%	NR NR NR NR	NR NR NR NR	100% 88% 86% 91%
71	Li	90.91% 87.1% 80% 89.56%	78.40% 84.64% 88.24% 76.47%	98.39% 76.47% 78.33% 89.79%	95% 92.1% 86.9% 90.5%
89	Nasrallah	NR NR NR NR NR NR NR NR	NR NR NR NR NR NR NR NR	NR NR NR NR NR NR NR NR	98% 88% 96% 84% 93% 90% 88% NR
93	Jin	88%	NR	NR	NR

3) Tumour identification and diagnosis

These studies involved classifying tumour types, such as metastatic spinal cord tumours, brain tumour subtypes, and pituitary adenomas, as well as paediatric tumour types (e.g., embryonal tumours vs. ependymomas). It includes differentiating between germinomas and craniopharyngiomas, distinguishing primary central nervous system lymphoma from atypical glioblastomas, and differentiating non-functioning pituitary adenomas from hypophysitis. Other highlights include the intra-operative cytological diagnosis of CNS tumours from squash smear slides and differentiation between tumefactive demyelinating lesions, gliomas, and CNS lymphomas. Additional areas involve identifying lytic spinal bone metastases, distinguishing pure from mixed germinoma pineal tumours, and detecting silent corticotroph adenomas. A total of 53 papers addressed these topics with 114 AI models validated on original data. The details are shown in Table 4

- Accuracy: The median accuracy was 91%, with an average of 88%, and a range of 42%.
- Specificity: The median specificity was 91%, with an average of 87%, and a range of 57%.
- Sensitivity: The median sensitivity was 89%, with an average of 86%, and a range of 42%.
- AUC: The median AUC was 91%, with an average of 89%, and a range of 38%.

**Table 4:** AI Performance in Tumour Identification and Diagnosis

Model number in spreadsheet	Study Purpose	First Author	Accuracy	Specificity	Sensitivity	AUC
2	To develop an AI model to differentiate between germinoma and craniopharyngioma	Chen	83% 82% 79% 77%	84% 84% 81% 82%	81% 80% 75% 71%	91% 91% 88% 88%
5	Differention between primary CNS lymphoma and atypical GBM	Han	83.70%	NR	NR	79.4%
6	To develop an AI model to differentiate between peripheral schwannomas from neurofibromas	Zhang	92.90% 83.3% 90.5% 78.6% 81% 81%	77.80% 77.8% NR NR NR NR	97% NR NR NR NR 91.03%	82.5% 92.3% 92.3% 87.2% 78.1% 91.4%
8	To evaluate the performance of an AI in differentiating GBM, brain metastases and primary CNS lymphoma	Tariciotti	94.65% 83.08% 81.07% 94.72%	96.23% 81.84% 88.46% 96.34%	91.03% 80.01% 63.61% 90.84%	98% 90% 81% 92%
10	To assess an AI’s ability to differentiate between non-functioning PitNETs and hypophysitis	Sahin	85% 58% 67% 61%	97% 52% 58% 52%	74% 64% 76% 70%	NR NR NR NR
11	To apply AI in distinguishing between paediatric supratentorial embryonal tumours, gliomas, and ependymomas	Zhang	89% 81% 91%	91% 69% 94%	85% 93% 82%	98% 82% 96%
12	To evaluate AI in classification of brain tumours	Stadlbauer	87.5% 83%	NR NR	NR NR	86.2% 88.6%
13	To develop an AI to differentiate between pilocytic astrocytomas from high-grade gliomas	Park	91.10% 91.1% 73.6%	93.10% NR NR	83.30% NR NR	93% 92% 79%
18	To evaluate an AI model using Raman Histology intra-operatively to diagnose Sino nasal and skull based tumours	Fitzgerald	93.30%	94.10%	93.30%	NR
19	To differentiate malignant brain tumours (GBM, primary CNS lymphoma, brain metastasis) using MRi data	Liu and Chen	76.90%	NR	NR	91%
21	AI model’s differentiation of GBM and metastases by MRI	Stadlbauer	91.20% 85.3%	NR NR	NR NR	91% 85%
22	To develop an Ai to detect and classify metastatic spinal cord compression on CTs	Hallinan	NR NR	95.47% 96.02%	93.38% 96.61%	94.4% 96.3%
24	To develop an AI that detects brain tumours using CNN	Joseph	93.40%	92.30%	92.30%	NR
26	To classify MB, EP and AP based on radiomics	Wang	93.80%	95.93%	91.87%	NC
28	To assess AI’s capability in intraoperative cytological diagnosis of CNS tumours	Ozer	95%	NR	Nr	98.5%
29	To evaluate AI’s performance in differentiating gliomas, PCNSL, and TPACNS	Miao	87%	NR	NR	90%
30	To investigate the diagnostic performance of an AI in GBM, lymphoma and metastasis	Joo	76.30%	84.43%	67.77%	87.8%
31	To evaluate the performance of an AI in differentiating between benign and malignant vertebral compression fractures	Liu and Jin	96.20%	99.20%	91.90%	98%
32	To develop an AI that detects brain metastases	Liew	NR NR NR NR NR NR	NR NR NR 94.5% 89% 91%	81.10% 74% 72.3% 82% 100% 67%	NR NR NR NR NR NR
35	To differentiate pilocytic astrocytomas from GBMs	Vats	NR NR	84.21% 63.16%	93.75% 93.75%	NR NR
39	To evaluate the performance of an AI in distinguishing between pure and non-germinoma pineal tumours	Supbumrug	88%	88%	81%	84.5%
41	To develop an AI model that detects and classifies lytic spinal bone metastases	Koike	87.20%	96.90%	74.10%	94.1%
42	To develop an AI model to differentiate between	Wang	83.85% 88.57%	90.96% 93.1%	65.28% 66.67%	93.1% 93.7%
44	To evaluate AI models in intra-operative brain tumour detection	Leon	86.80% 91.5%	90% 90%	65.90% 57.8%	NR NR
48	To develop an AI that differentiates between cerebral cystic metastases from brain abscesses	Cui	97% 96%	93% 93%	100% 100%	100% 100%
49	To develop an AI for differentiating between solitary fibrous tumours from angiomatous meningiomas	Li	97.10% 94.1% 88.2%	100% 94.7% 94.7%	93.30% 93.3% 80%	98.9% 96.78% 91.1%
51	To develop an AI for intraoperative tissue classification of gliomas through OCT imaging	Fischer	NR	86.30%	86.70%	NC
52	To evaluate an AI’s ability to differentiate different types of brain tumours	Liu ad Wang	99.47% 95.07%	NR NR	NR NR	NR NR
53	To develop an AI for the detection of gliomas, meningiomas and pituitary tumours	Priya	97%	NR	96.78%	NR
55	To develop an AI that differentiates between supratentorial extraventricular ependymoma from supratentorial GBM	Chen	82.50%	88.90%	69.20%	79.6%
56	To develop an AI that differentiates between cancer, meningioma, schwannomas	Haim	78%	NR	NR	Nr
57	To develop AI models that distinguish between intraspinal schwannomas from meningiomas	Xu	81.40% 86% 81.1% 81.1%	66.70% 90% 71.4% 81%	89.30% 83.9% 87.5% 81.2%	89% 95.6% 83.8% 92.2%
60	Classification of GBM, intracranial metastatic disease, and primary CNS lymphoma	Bathla	NR NR NR NR NR	NR NR NR NR NR	NR NR NR NR NR	73% 64% 66% 67% 68%
61	Development of an AI that detects spinal metastasis	Mostafa	98.20%	97.80%	100%	NR
62	To test an AI’s ability to discriminate between tuberculomas, gliomas and metastasis	Indoria	92.30% 88% 82%	NR NR NR	NR NR NR	96% 93% 90%
64	To develop an AI to differentiate between high-grade gliomas and solitary brain metastases	Xiong	92.78% 89.66% 91.11% 90%	90% 83.33% 95.55% 86.66%	95.55% 90.91% 87.78% 93.33%	NR 93.9% 86.6% 82.6%
65	To investigate an effectiveness of AI in distinguishing between primary CNS lymphoma and GBM	Yang	NR NR NR NR	NR NR NR 78%	NR NR NR 93%	86% 91% 89% 92%
67	To develop an AI for detection and classification of brain tumours	Nawaz	99.13% 99.08%	NR NR	97.25% 97.14%	NR NR
69	To develop an AI to differentiate filum terminale ependymomas from schwannomas	Gu	87%	81%	88%	93%
72	To develop an AI for classification of brain tumours using MRIs	Tonmoy	99.30%	NR	99.30%	NR
73	To develop an AI for detecting brain metastases using MRI scans	Zhou	NR	NR	81%	NR
78	To develop an AI to detect small and subtle brain metastases	Fairchild	NR	NR	89%	NR
82	To develop an AI for classification of brain tumours	Saeedi	90.92%	NR	94.25%	NR
84	To develop an AI for classification of tumours	Tandel	100% 95.97% 96.65% 87.14% 93.74%	100% NR NR NR NR	100% NR NR NR NR	100% 99% 100% 98% 99%
85	To develop an AI with near-infrared fluorescence imaging for intra-operative glioma diagnosis	Shen	NR 87.5% NR	82.20% 90.9% 43.4%	93.80% 60.6% 77.8%	94.5% 81% 62.5%
86	To develop an AI for early brain tumour diagnosis using MRI images	Senan	95.10% 91.2% 93.3% 93.8%	98.50% 97% 97.5% 97.5%	95.25% 91.5% 93% 93.75%	NR NR NR NR
87	To develop an AI that classifies gliomas, meningiomas, and pituitary tumours	Patil	97.77%	98.33%	96.66%	NR
90	To develop an AI for classifying brain tumours in MRI	Mohan	98.78% 93.9%	NR NR	98.78% 93.9%	99.98% 95.41%
91	To develop an AI for brain tumour classification between meningiomas, gliomas and pituitary tumours	Maqsood	97.47% 98.92%	97.94% 99.02%	97.22% 98.82%	NR NR
94	To develop an AI using raman histology to analyse skull base tumour specimens intra-operatively	Hollon	91.50% 83.1% 96.6%	NR NR NR	NR NR NR	NR NR NR
95	To develop an AI using Raman histology to predict brain tumour diagnosis intra-operatively	Hollon	94.60%	NR	NR	NR
96	To develop an AI to identify brain tumours from MRI data	Gao	73.30%	96.30%	88.90%	92%

4) Tumour segmentation and delineation

Some papers investigated segmentation of craniopharyngiomas and their subtypes, intra-operative brain tumour boundary detection to maximize resection, and segmentation of lumbar spine stenosis. Segmentation of pituitary adenomas, posterior fossa ependymomas, and pituitary macroadenomas with cavernous sinus invasion was also explored. Additionally, volumetric measurement of vestibular schwannomas and segmentation of brain, skin, tumours, and ventricles were addressed. This topic was covered in 23 papers, with a total of 50 AI models or original datasets assessed. Details are shown in Table 5

- DSC: The median DSC was 87%, with an average of 81%, and a range of 95.1%.
- HD: The median HD was 3.12 mm, with an average of 3.65 mm, and a range of 11.50 mm.
- VEE: The median VEE was 34%, with an average of 46%, and a range of 69%.
- ICC: The median ICC was 0.89, with an average of 0.89, and a range of 0.

**Table 5:** AI Performance in Tumour Segmetation and Delineation

Model number in spreadsheet	Study Purpose	First Author	DSC	HD	VEE	ICC
17	To assess AI performance in tumour segmentation based on MRI biomarkers in brain metastasis	Jalalifar	0.87	2.74 mm	15.90%	NR
25	To develop Ai capable of segmentation and classifying craniopharyngiomas	Yan	0.8668	NR	NR	NR
36	to develop an AI to segment high grade gliomas, metastases and meningiomas	Cekic	0.91 0.89	NR NR	NR NR	NR NR
37	To evaluate several AI’s performance in tumour contour segmentation	Jalalifar	0.81 0.82 0.87	3.7 mm 3.5 mm 2.74 mm	21.50% 18.9% 15.9%	NR NR NR
45	To evaluate an AI that segments pituitary adenomas	Faern	88.9% 91%	NR NR	NR NR	NR NR
46	To develop an AI that provides automated volumetric segmentation for pituitary adenomas	Mutten	0.62 0.046	3.89 mm 12.199 mm	85% 22%	NR NR
68	To develop an AI for segmentation of pituitary macroadenomas and cavernous sinus invasion	Rui	0.91	2.13 mm	NR	NR
70	To develop an AI model to segment brain metastasis	Luo	0.91	NR	NR	NR
74	To evaluate an AI’s ability to segment metastatic brain tumours	Wang	0.723	0.704 mm	NR	NR
75	To develop an AI that quantifies brain metastases	jeong	NR	NR	NR	0.893
76	To develop an AI model that segments brain tumours	Jalalifar	0.88	1.25 mm	NR	NR
77	To evaluate the performance of an AI in volumetrically analysing brain metastases	Hammer	0.87	NR	NR	0.893
79	To develop an AI model for segmenting brain tumours intraoperatively using thermal infrared imaging	Cardone	0.798 0.723 0.904 0.904	NR NR NR NR	30.87% 63.49% 36.65% 84.64%	NR NR NR NR
80	To evaluate the performance of an AI in brain metastases segmentation	Bousabarah	NR NR	NR NR	82% 77%	NR NR
83	To develop an AI for delineation of brain metastases	Zhao	0.883	NR	NR	NR
92	To develop an AI to volumetrically measure vestibular schwannoma following gamma knife radiosurgery	Lee	0.9 0.87	NR NR	NR NR	NR NR
97	To evaluate AI in the segmentation of brain, skin, tumours and ventricles in MRI scans	Boer	0.971 0.936 0.997 0.991 0.926 0.723 0.91 0.856	NR NR NR NR NR NR NR NR	NR NR NR NR NR NR NR NR	NR NR NR NR NR NR NR NR
98	To evaluate AI in segmentation of brain tumours via US	Carton	0.47 0.61 0.65	NR NR NR	NR NR NR	NR NR NR
99	To develop a real-time brain tumour detection system using the YOLO11 architecture on intraoperative ultrasound (ioUS)	Cepeda	0.87 0.88 0.88 0.87 0.88	NR NR NR NR NR	NR NR NR NR NR	NR NR NR NR NR
100	To evaluate a semi-automatic segmentation method to extract brain tumor contours from 3D intraoperative ultrasound (iUS) images	Angel-Raya	0.87 0.81 0.70	NR NR NR	NR NR NR	NR NR NR
101	To train a deep learning model for automatic brain tumor segmentation in 2D iUS images	Faanes	0.58 0.62 0.59	NR NR NR	NR NR NR	NR NR NR
102	To use 2D and 3D U-Net networks to segment the resection cavity in intraoperative ultrasound (iUS) images	Carton	0.72	NR	NR	NR
103	To develop and evaluate NeuroIGN, an, AI-driven system for precise brain tumor surgery using real-time image guidance	Zeineldin	0.93	3.6 mm	NR	NR

##### Vascular neurosurgery

A total of 36 vascular neurosurgical conditions were categorized for clarity and relevance. One study was placed across 2 categories. Such categories include.

1. Stroke detection

This includes detecting stroke lesions (DWI), classifying strokes, triaging major ischemic strokes, and using CT imaging for acute cerebral infarction. These studies also covered detection of large vessel occlusions and cerebral venous thrombosis. In total, 10 studies were included with 24 AI models or original data sets evaluated. Details are shown in Table 6.

- Accuracy: The median accuracy was 89%, with an average of 86%, and a range of 45%.
- Specificity: The median specificity was 88%, with an average of 84%, and a range of 54%.
- Sensitivity: The median sensitivity was 84%, with an average of 86%, and a range of 30%.
- AUC: The median AUC was 88%, with an average of 85%, and a range of 31%

**Table 6:** AI Performance in Stroke Detection

Model number in spreadsheet	Purpose	First Author	Accuracy	Specificity	Sensitivity	AUC
3	To develop a deconvolution AI based CT perfusion imaging system that diagnoses acute cerebral infarction	Chen	93.70%	98.70%	NR	NR
			87.60%	93.20%	NR	NR
8	To evaluate the performance of whole brain dynamic radiomics features for stroke detection	Guo	NR	NR	NR	73.10%
			NR	NR	NR	65.20%
			NR	NR	NR	87.30%
			NR	NR	NR	79.50%
			NR	NR	NR	
9	To develop an AI for classifying strokes	Yalçın	98.90%	NR	98.80%	NR
			98.80%	NR	98.70%	NR
			98.60%	NR	98.50%	NR
			98.31%	NR	98%	NR
10	To evaluate an efficient stroke screening AI for emergency departments	Cai	71.40%	45.16%	81.13%	71.63%
11	To develop an AI to detect stroke lesions from DWI	Chen	NR	NR	72.88%	NR
			NR	NR	74.44%	NR
			NR	NR	75.80%	NR
13	To develop an AI model during the triage of major ischemic strokes	Desai	NR	NR	83.90%	93.10%
14	To evaluate the accuracy of an AI for detecting large vessel occlusions	Matsoukas	91.20%	91.10%	93.80%	95%
			89.80%	91.10%	74.60%	86%
18	To evaluate an AI for cerebral venous thrombosis detection	Yang	NR	88%	96%	96%
28	To evaluate the performance of an AI in classifying AIS	Oura	54%	79%	81%	76%
			74%	79%	81%	91%
			80%	85%	69%	94%
32	To evaluate the performance of an AI in detecting strokes on MRI scans in the ER	Kim	89%	89%	90%	95%
			83%	84%	89%	88%

2) Intracranial haemorrhage detection

These studies focused on detecting ICHs, nontraumatic subarachnoid haemorrhages, the segmentation of aneurysmal subarachnoid haemorrhages, and the detection of intracranial hematomas. In addition, there were also studies which classified ICH subtypes, detected cerebral microbleeds and the occlusions of intracranial arteries. In total there were 13 studies with 22 AI models or original data sets evaluated. Details are shown in Table 7

- Accuracy: The median accuracy was 91%, with an average of 87%, and a range of 31%.
- Specificity: The median specificity was 94%, with an average of 91, and a range of 51%.
- Sensitivity: The median sensitivity was 90%, with an average of 88%, and a range of 36%.
- F1 Score: The median F1 score was 87%, with an average of 84%, and a range of 38%
- AUC: The median AUC was 97%, with an average of 94%, and a range of 14%

**Table 7:** AI Performance in in Aneurysm Diagnosis

Model number in spreadsheet	Purpose	First Author	Accuracy	Specificity	Sensitivity	F1 score	AUC
2	To develop an AI that detects intracerebral haemorrhages	Yu	NR	99.90%	80.70%	86.10%	NR
			NR	99.90%	87.60%	87.40%	NR
4	To evaluate the performance of an AI for detection of intracranial haemorrhage	Seyam	93%	93.90%	87.20%	78%	NR
7	To evaluate the RAPID ICH software in detecting intracranial haemorrhage	Eldaya	85.30%	84.40%	91.90%	60.20%	NR
12	To develop an Ai for segmentation of intracerebral haemorrhages	Nijiati	91.25%	84%	98.50%	NR	NR
16	To evaluate the performance of an AI for identifying occlusions and intracranial haemorrhage	Yedavalli	NR	99.50%	96.20%	NR	NR
			NR	95.10%	63.50%	NR	NR
21	To develop an AI model for the detection of haemorrhages	Hibi	79%	78%	85%	NR	89%
			67%	49%	96%	NR	92%
24	To develop an AI to detect intracranial hematomas	El Refaee	NR	94%	NR	NR	NR
			NR	93%	NR	NR	NR
26	To evaluate AI performance in automatic detection of intracranial haemorrhages	Yeo	NR	NR	NR	NR	85.40%
			NR	NR	NR	NR	96.60%
			NR	NR	NR	NR	97%
			NR	NR	NR	NR	96.60%
27	To validate the performance of an AI to detect ICH and it’s subtypes	Neves	98.05%	100%	97.52%	98.63%	98.76%
30	To develop an AI that segments aneurysmal subarachnoid haemorrhages	Garca	NR	99.70%	72.30%	NR	NR
			NR	99.60%	82.10%	NR	NR
			NR	85.50%	85.50%	NR	NR
33	To validate the performance of an AI in detecting cerebral microbleeds	Won	NR	NR	95.20%	NR	NR
36	To assess an AI model’s ability to assess intracranial haemorrhages	Voter	NR	97.70%	92.30%	86.50%	NR
38	To develop an AI which detects nontraumatic subarachnoid haemorrhages	Nishi	95%	92%	99%	94%	99%

3) Aneurysm diagnosis: These studies focused on detecting ICAs, such as saccular, ruptured, and unruptured aneurysms. Some studies also explored the segmentation of ICAs and intracranial haemorrhages. In total there are 14 studies with 25 AI models or original data sets evaluated. Details are shown in Table 8
  Accuracy: The median accuracy was 88%, with an average of 89%, and a range of 3%.
  Specificity: The median specificity was 92%, with an average of 90%, and a range of 23%.
  Sensitivity: The median sensitivity was 88%, with an average of 88%, and a range of 61%.
  Dice Score: The median DS was 81%, with an average of 76%, and a range of 50%
  AUC: The median AUC was 85%, with an average of 83%, and a range of 16%

**Table 8:** AI Performance in Aneurysm Diagnosis

Model number in spreadsheet	Study Purpose	First Author	Accuracy	Specificity	Sensitivity	Dice score	AUC
5	To validate the performance of an AI in detecting ICA’s	Calasurdo	88.30%	94.20%	81.70%	NR	NR
6	To develop an AI model to detect intracranial aneurysms in digital subtraction images	Ou	NR	NR	80.06%	83.16%	NR
15	To develop an AI to detect saccular aneurysms	Claux	NR	NR	78%	NR	NR
17	To develop an AI model that detects unruptured cerebral aneurysms	Chen	NR	NR	82.46%	NR	NR
19	To develop an AI for detection of cerebral aneurysms	Dai	NR	NR	91.80%	NR	NR
20	To evaluate an AI that detects IA’s from CTA scans	Wang	NR NR NR NR NR	NR NR NR NR NR	89.20% 94.2% 92.5% 97% 95.7%	NR NR NR NR NR	NR NR NR NR NR
22	To evaluate an AI for the identification of aneurysms	Hu	NR	NR	94.50%	NR	NR
23	To evaluate Ai’s performance in aneurysm detection	Toledo	NR	97.60%	36%	41.14%	NR
25	To develop an AI to detect ruptured and unruptured aneurysms	Feng	NR NR NR	NR NR NR	NR NR NR	NR NR NR	85% 88% 86%
29	To assess the performance of an AI in detecting ICAs	Joo	88.30% 91.3%	92% 98%	87.10% 85.7%	89.80% 91.5%	NR NR
31	Detection of unruptured intracranial aneurysms	Li	91.86%	96%	91.63%	NR	96%
34	To evaluate an AI model’s classification of intracranial haemorrhages	Angkurawaranon	NR NR NR	NR NR NR	NR NR NR	NR NR NR	85% 83% 72%
35	To evaluate AI performance in detection and segmentation of of ICAs	You	NR	NR	96.40%	78.30%	NR
37	To develop an Ai for the detection of ICAs	Shi	NR NR NR	74.70% 80.2% 89.7%	97.30% 84.6% 78.6%	75% NR NR	NR NR NR

### Functional neurosurgery

These studies were categorised for clarity and relevance. A total of 37 studies were included.

1. Parkinson’s Diseases

These studies examined AI's role in diagnosing Parkinson's disease and identifying associated axial postural abnormalities, including Pisa Syndrome and thoracic and lumbar Camptocormia. Additional research focused on differentiating PD from other conditions such as dementia with Lewy bodies, cerebellar ataxia, multiple system atrophy, spinocerebellar degeneration, and progressive supranuclear palsy Richardson syndrome. In total there were 11 studies and 13 AI models or original data sets evaluated. Details are provided in Table 9

- Accuracy: The median accuracy was 94%, with an average of 89%, and a range of 29%.
- Specificity: The median specificity was 94%, with an average of 94%, and a range of 17%.
- Sensitivity: The median sensitivity was 84%, with an average of 85%, and a range of 44%.
- AUC: The median AUC was 97%, with an average of 94%, and a range of 22%
- ICC: The median ICC was 0.97, with an average of 0.97, and a range of 0.00.

**Table 9:** AI Performance in Parkinson's Disease Diagnosis

Model number in spreadsheet	Purpose	First Author	Accuracy	Specificity	Sensitivity	AUC	ICC
3	To develop an AI that differentiates between Parkinson’s, cerebellar ataxia and progressive supranuclear palsy Richardson syndrome	Song	NR NR NR	96.90% 93.2% 93.6%	82.90% 82.3% 55.5%	97.40% 97.5% 96.3%	NR NR NR
5	To develop an Ai that differentiates PD from normal controls	Sun	94.38%	96.94%	88.77%	NR	NR
9	To automatically diagnose PD	Li	93.50%	100%	87.10%	97.60%	NR
12	To assess an AIs ability to assess axial postural abnormalities of Parkinsonism’s	Artusi	NR	NR	NR	NR	97%
13	To develop an AI’s diagnostic accuracy of PDs	Yang	96.10%	97.40%	95.20%	NR	NR
15	To provide differential diagnosis of Parkinsonism	Tsai	84.10%	83.20%	84.60%	NR	NR
17	To segment and detect PD from MRI scans	Tassew	96%	NR	98%	NR	NR
18	To develop an AI that aids in diagnosis of PD, PSP, and MSA	Kim	84%	94%	84%	NR	NR
21	To differentiate Parkinson’s from spinocerebellar degeneration	Eguchi	87%	89%	83%	93%	NR
23	To develop an Ai to assess videos of finger tapping to classify idiopathic PD vs healthy controls	Yang	69%	NR	76%	76%	NR
26	To develop an AI for PD diagnosis using speech datasets	Elkharadly	97.73%	NR	99%	98%	NR

2) Dementia: AD, dementia with Lewy bodies, the studies also involve AI’s diagnosis of MCI relative to dementia and healthy controls. In total there were 6 studies, and 14 AI models or original data sets evaluated. Details are provided in Table 10
  Accuracy: The median accuracy was 92%, with an average of 92%, and a range of 14%.
  Specificity: The median specificity was 85%, with an average of 77%, and a range of 55%.
  Sensitivity: The median sensitivity was 95%, with an average of 94%, and a range of 12%.
  AUC: The median AUC was 86%, with an average of 86%, and a range of 23%.

**Table 10:** AI Performance in Dementia Diagnosis

Model number in spreadsheet	Purpose	First Author	Accuracy	Specificity	Sensitivity	AUC
4	To develop an AI to help diagnose AD	Fujita	NR NR	50.40% 45.3%	95.40% 94%	81.90% 81.7%
7	To develop an AI for diagnosing Parkinson’s and dementia with Lewy bodies	Nakajima	NR NR NR	NR NR NR	NR NR NR	86% 88% 93%
8	To evaluate an AI’s diagnostic performance in ADs and mild cognitive impairment	Deng	88.41% 92.07% 96.95%	93.75% 97.92% 100%	92.24% 95.69% 97.41%	NR NR 98%
10	To develop an AI that detects ADs and MCI compared to healthy controls	Shi	NR NR	NR NR	NR NR	86.70% 75.2%
19	To assess the performance of seed amplification assay and raman spectroscopy in the diagnosis of AD	D'Andrea	85%	77%	88%	85%
20	To assess an AI’s ability to distinguish between other neurological conditions and AD	Assaduzzaman	98.78% 97.5% 87.11%	NR NR NR	98% 97% 86%	NR NR NR

3) Epilepsy

These studies focus on detecting epilepsy, determining seizure onset lateralization, and distinguishing between temporal-plus and temporal lobe epilepsy. Other studies differentiate generalized tonic–clonic, focal to bilateral tonic–clonic, and nonconvulsive seizures. Some studies also identified epilepsy types in specific regions, such as the temporal lobe, frontal lobe, and perirolandic areas. Studies also examined temporal lobe epilepsy subtypes, including focal awareness seizures, focal impaired awareness seizures, and focal to bilateral tonic–clonic seizures. In total there were 12 studies, with 15 AI models or original data sets evaluated. Details are provided in Table 11

- Accuracy: The median accuracy was 92%, with an average of 90%, and a range of 23%.
- Specificity: The median specificity was 94%, with an average of 87%, and a range of 32%.
- Sensitivity: The median sensitivity was 93%, with an average of 89%, and a range of 21%.
- AUC: The median AUC was 94%, with an average of 89%, and a range of 20%.

**Table 11:** AI Performance in Epilepsy Detection

Model number in spreadsheet	Purpose	First Author	Accuracy	Specificity	Sensitivity	AUC
2	Ai detection of interictal epileptiform discharges	Geng	96.26%	96.47%	95.85%	96.40%
11	To assess AI’s ability to lateralise seizure onset in epileptic patients	Kaestner	78%	NR	77.40%	85.70%
14	Classification of epileptic seizure types	Alshaya	97%	97%	98%	NR
16	To differentiate temporal-plus epilepsy from temporal lobe epilepsy	Yin	87.50%	92.90%	80%	77.90%
24	EEG interpretations that indicate epilepsy, encephalopathy and focal brain lesions	Mansilla	92%	94%	88%	NR
25	To classify temporal lobe epilepsies	Gleichgerrcht	74.57%	NR	NR	NR
27	To develop an AI that identifies the epileptogenic side in patients with mesial temporal lobe epilepsy	Yu	92.40%	NR	NR	97%
28	An AI for detection and localisation of epileptic sources	Zheng and Liao	87.18%	NR	NR	Nr
29	To detect seizures using wearable AI biosensors	Yu	NR NR	64.70% 68.3%	83.90% 77.2%	78.90% 76.90%
30	Seizure detection for focal-onset seizures	You	NR NR	NR NR	96.30% NR	93.72% 93.93%
31	To detect focal-onset seizures in the anterior nucleus of thalamus	Toth	93.90% 97.3%	NR NR	93.80% 97.6%	NR NR
32	Seizure and epilepsy zone detection	Bhanot	93.40%	93%	93%	97%

4) Multiple Sclerosis: These studies assessed AI’s performance in delineating white matter lesions in MS, and AI differentiation between MS and cerebral small vessel disease. In total there were 2 studies and 5 AI models or original data assessed. Details are provided in table Table 12
  Accuracy: The median accuracy was 77%, with an average of 65%, and a range of 65%.
  Specificity: The median specificity was 89%, with an average of 89%, and a range of 0.0%.
  Sensitivity: The median sensitivity was 87%, with an average of 87%, and a range of 20%.
  DSC: The median DSC was 86%, with an average of 82%, and a range of 36%.

**Table 12:** AI Performance in Multiple Sclerosis Detection

Model number in spreadsheet	First Author	Accuracy	Specificity	Sensitivity	DSC
6	Hindsholm	NR	NR	77%	62%
22	Xu	86.06% 83.19% 20.65% 71.6%	89.17% NR NR NR	96.89% NR NR NR	97% 98% 66% 86%

### Spinal neurosurgery

1. Spinal Diseases

These studies examined AI's role in diagnosing patients with central lumbar stenosis, detection, and classification of disc herniation. Classification of osteoporotic and non-osteoporotic vertebral fractures. Diagnosis of lumbar spondylolisthesis. Diagnosis of lumbar disc herniation with L5 and S1 radiculopathy. Detection of cervical spine fractures. In total there were 17 studies with 37 AI models or original data sets evaluated. Details are provided in Table 13

- Accuracy: The median accuracy was 91%, with an average of 86%, and a range of 35%.
- Specificity: The median specificity was 97%, with an average of 95%, and a range of 16%.
- Sensitivity: The median sensitivity was 89%, with an average of 85%, and a range of 34%.
- AUC: The median AUC was 89%, with an average of 93%, and a range of 39%.

**Table 13:** AI Performance in Spinal Disease Detection

Model number in spreadsheet	Purpose	First Author	Accuracy	Specificity	Sensitivity	AUC
2	To triage patients with central lumbar stenosis	Kim	82.80%	84.60%	81%	90%
3	To develop an Ai that classifies Amyotrophic Lateral Sclerosis patients and healthy controls	Bede	75.40% 74.3% 78.5%	NR NR NR	82.50% 83.6% 79.1%	93% 83.5% 90.7%
4	To produce an AI which classifies lumbar disc herniations	Tijana	80%	NR	NR	NR
5	To localise nerve root compression in lower lumbar disc herniations	Wang	84%	NR	84%	93%
6	Performance of an AI for insufficiency fracture detection	Germann	96.20%	96.90%	94.10%	NR
9	AI localisation and classification of intervertebral disc herniation	Valarmathi	93.59% 88.46% 84.62%	96.97% NR NR	91.11% NR NR	NR NR NR
11	To classify osteoporotic vertebral fractures	Zhang	NR NR	NR NR	NR NR	91.30% 83.1%
12	AI diagnosis of fresh vertebral fractures	Wang	97.60% 92.4%	97% 92%	98% 93%	98% 9%
13	Diagnosis of lumbar spondylolisthesis	Xu	92.30% 93.1% 92.3% 89.2% 90.8%	NR NR NR NR NR	93.10% 93.6% NR NR NR	93.40% NR 93.4% 91.4% 96.5%
14	To detect osteoporotic compression fracture detection	Liawrungrueang	96.04%	94.28%	95.85%	96.50%
15	Diagnosis of lumbar disc herniation of L5 and S1 radiculopathy	Wang	89.50% 78.81% 80.99% 63.37%	NR NR NR NR	94.05% 89.88% 89.29% 78.87%	94.85% 86.75% 87.94% 58.6%
16	Detection of cervical spine fractures	Liawrungrueang	92.14%	95.70%	88.60%	92.10%
17	Vertebral fracture detection	Chou	93.36% 93.36% 89.41% 96.23% 95.74% 92.26%	94.26% 98.49% 89.91% 97.67% 96.85% 99.07%	88.97% 65% 86.92% 72.22% 89.95% 63.73%	NR NR NR NR NR NR
18	Predication of thoracolumbar injury classification	Doerr	86.8.%	NR	90%	NR
21	AI detection of fractures in cervical spine CTs	Wittenboer	96.10%	98.60%	71.50%	NR
22	Diagnosis of osteoporotic vertebral fractures	Shen	96.10% 98.2%	98.60% 99.2%	71.50% 88.2%	NR NR
23	Diagnosis of traumatic thoracolumbar fractures on saggital radiographs	Rosenberg	88% 86%	89% 83%	91% 90%	NR NR

2) Spinal Segmentation

Segmentation of spinal stenosis and intervertebral disc herniation, and lumbar disc segmentation, AI in measurement of full alignment parameters in whole-spine lateral radiographs. In total there were 7 studies included and 13 AI models or original datasets assessed. Details are provided in Table 14

- IoU: The median was 90%, with an average of 89%, and a range of 15%.
- Dice Score: The median Dice Score was 89%, with an average of 89%, and a range of 10%.
- HD: The median HD was 11.7 mm, with an average of 11.78 mm, and a range of 3.8 mm.
- ASD: The median ASD was 0.18 mm, with an average of 0.16 mm, and a range of 0.10 mm

**Table 14:** AI Performance in Spinal Segmentation

Model number in spreadsheet	Purpose	First Author	IoU	Dice Score	HD	Average Surface Distance
7	Diagnosis and segmentation of lumbar spine stenosis	Altun	98% 90% 86%	NR NR NR	NR NR NR	NR NR NR
8	To segment the lumbar spinal canal in cases of lumbar stenosis	Laiwalla	NR NR	88% 89%	11.7 mm 13.1 mm	0.18 mm 0.18 mm
9	AI localisation and classification of intervertebral disc herniation	Valarmathi	89.64%	NR	NR	NR
10	Segmentation of lumbar disc MRI images	He	NR	84.05%	NR	NR
19	AI measurement of full alignment parameters in whole spine lateral radiographs	Landriel	83%	NR	NR	NR
20	Detection of cervical spinal cord compression	Laiwalla	NR	88% 89% 94%	11.7 mm 13.1 mm 9.3 mm	0.18 mm 0.18 mm 0.08 mm

## Discussion

AI across various neurosurgical subfields highlight potential future applications, with many studies reporting median accuracies well above 85%.

In Neuro-Oncology where almost half of the papers were classified into both classification tasks (e.g., normal vs. abnormal tissue) and tumour subtyping or grading reliably assisted in distinguishing tumour grades. Segmentation tasks, though robust in terms of Dice Similarity Coefficients and low Hausdorff Distances, indicate some variability, underscoring the technical challenges of precisely delineating complex intracranial structures.

Similar trends emerge in Vascular Neurosurgery, where models tasked with stroke detection, intracranial haemorrhage identification, and aneurysm diagnosis. Many AI models demonstrate high specificity, often around 90% or higher in some studies, suggesting potential for reducing unnecessary follow-up imaging. However, clinical validation is needed to assess their real-world impact. Notably, ICH detection also exhibits impressive AUC metrics in many papers with a median of 97%, “underscoring potential future applications.”

In Functional Neurosurgery, the focus ranges from diagnosing Parkinson’s disease and dementia subtypes to localizing seizure foci in epilepsy. Reported accuracies routinely hover around the 90% mark or above, with some studies exceeding 94%. However, these findings often come from relatively small datasets, indicating a need for larger heterogeneous studies.

Finally, Spinal Neurosurgery applications—including detection and classification of lumbar disc herniation, vertebral fractures, and spinal stenosis—also show high accuracy. Specificities often exceed 90%, reflecting AI’s aptitude for ruling out false positives in spinal pathologies. Automated segmentation techniques for spinal alignment parameters or intervertebral discs similarly display encouraging results, though consistent accuracy in clinical practice demands further validation.

Some AI models have shown diagnostic performance comparable to, and in certain cases exceeding, that of expert radiologists and neurosurgeons, though real-world clinical integration remains a challenge. They can save doctors time, but also present legal liability considerations. In this sense, AI is more akin to another technological advance – such as the MRI—rather than a replacement for diagnostic neurosurgery.

Most of the AI models in these studies were hybrid models, a sign of early innovation that may eventually narrow to a few standard types. The limitations of this review include the lack of data weighting and absence of a meta-analysis. Additionally, the study's data collection was limited to early 2025, potentially missing newer publications. Variability in study design and quality contributed to inconsistencies in performance metrics, and some studies had a higher risk of bias.

## Clinical translational relevance

AI may one day enhance preoperative planning, intraoperative guidance, and postoperative monitoring. However, challenges remain, including clinician training, regulatory approval, and integration into existing workflows. Addressing issues such as AI interpretability and surgeon trust in AI-driven recommendations is crucial for widespread adoption. Collaboration between clinicians, engineers, and data scientists will be key to overcoming these barriers.

## Future perspectives

Future AI advancements should prioritise prospective, multicentre studies for validation, integrating ultrasound with MRI/CT for broader applicability, and addressing rare conditions (e.g., atypical or mycotic aneurysms). Standardised datasets, interoperability across institutions, and real-world clinical testing will be essential for regulatory approval and widespread clinical adoption.

## Limitations

The study's limitations include the absence of data weighting, as it is not a systematic review with a meta-analysis. Instead, its purpose is to provide a broad overview of the current state of AI as of early 2025. This study also has a limited number of references as the focus was to provide a compilation of data. In addition, this paper has a broad scope, hence limiting statistical precision. The timeframe (January 2020–January 2025) may exclude newer studies beyond early 2025. The high volume of studies included also lead to an inevitable variability in study quality (e.g., small datasets, retrospective designs) and overfitting. Exclusion of non-English/Chinese studies may have skew findings, potentially missing relevant global research, particularly Korean, German and Indian language studies which have substantial AI research in medicine.

## Conclusion

Conclusion: AI offers promising potential for improving diagnostic accuracy in neurosurgery, with median accuracies above 85% in neuro-oncology (e.g., 91% for tumour identification), vascular (e.g., 97% AUC for ICH), functional (e.g., 94% for Parkinson’s), and spinal applications (e.g., 91% for diseases). However, sometimes significant variability in sensitivity, specificity, and AUC across studies suggests a need for standardized datasets and real-world validation to ensure clinical reliability.

Future work should focus on standardising datasets, improving model robustness, and conducting real-world validation to ensure AI technologies are clinically viable and effective in neurosurgical practice.

## Supplementary Information

Below is the link to the electronic supplementary material.

Supplementary file1 (CSV 188 KB)

Supplementary file2 (CSV 38 KB)

Supplementary file3 (CSV 62 KB)

Supplementary file4 (CSV 45 KB)

## Data Availability

No datasets were generated or analysed during the current study.
